# The Correlation of Surgeon Subspecialty With Outcomes Following Surgery for Geriatric Femoral Neck Fracture

**DOI:** 10.5435/JAAOSGlobal-D-25-00103

**Published:** 2025-09-18

**Authors:** Justin R. Zhu, Ismail Ajjawi, Wesley Day, Michael J. Gouzoulis, Anthony Seddio, Jonathan N. Grauer

**Affiliations:** From the Department of Orthopaedics and Rehabilitation, Yale School of Medicine, New Haven, CT.

## Abstract

**Introduction:**

Geriatric femoral neck fractures are common and typically managed with hemiarthroplasty (HA), total hip arthroplasty (THA), or percutaneous pinning (PP) by an on-call physician. The current study assessed if perioperative or longer-term outcomes correlated with orthopaedic surgeon subspecialty.

**Methods:**

The 2016 to 2022 PearlDiver M161 Ortho administrative data set was used to identify patients >65 years who underwent HA, THA, or PP for femoral neck fracture. The subspecialty of the treating surgeon was defined as arthroplasty, trauma, or nonarthroplasty/nontrauma. Exclusion criteria included polytrauma and concurrent neoplasms/infections. For each surgery type, 90-day perioperative adverse events were assessed between surgeon cohorts using multivariate logistic regression. Five-year revisions/dislocations were also assessed.

**Results:**

Overall, 150,728 surgeries were identified (140,850 by nontrauma/nonarthroplasty surgeons, 5,013 arthroplasty, and 4,865 trauma). Arthroplasty surgeons performed THA at higher rates than trauma or nonarthroplasty/nontrauma surgeons (28.1% versus 7.7% and 12.8%, respectively, *P* < 0.001). Ninety-day outcomes were more similar than different across surgeon specialties. For HA, 5-year dislocation rates were statistically different but within 1% between cohorts (nonarthroplasty/nontrauma 97.5%, arthroplasty 96.8%, trauma 97.8%). Five-year revision rates were also within 1% between the cohorts (nonarthroplasty/nontrauma 98.2%, arthroplasty 97.2%, trauma 97.8%). For THA, 5-year dislocation rates were not statistically different (nonarthroplasty/nontrauma 95.7%, arthroplasty 96.3%, trauma 96.9%), nor were five-year revision rates (nonarthroplasty/nontrauma 93.9%, arthroplasty 93.8%, trauma 95.0%). For PP, 5-year revision rates were not statistically different (nonarthroplasty/nontrauma 93.8%, arthroplasty 95.1%, trauma 95.2%).

**Conclusion:**

Femoral neck fractures were predominantly treated by nonarthroplasty/nontrauma surgeons. Nonetheless, 90-day adverse outcomes and five-year rates of revision/dislocation were clinically quite similar. This can provide confidence that those who self-select to treat geriatric femoral neck fractures are performing comparably regardless of subspecialty.

Geriatric femoral neck fractures are common injuries with notable related morbidity, mortality, and effect on health-related quality of life.^1,2^ In the United States alone, more than 300,000 older adults are hospitalized for hip fractures each year, and this number is expected to grow with an aging population.^2^ These injuries carry a one-year mortality rate of up to 30%.^2^ In the United Kingdom, more than 75,000 hip fractures occur annually, representing a similar trend and substantial healthcare burden.^2^ These are typically managed surgically with hemiarthroplasty (HA), total hip arthroplasty (THA), or percutaneous pinning (PP) in an expedited fashion by an on-call orthopaedic physician who may be of variable subspecialty.^3-5^

Subspecialization following graduation from orthopaedic surgery residency remains the most common next step for trainees, pursued by nearly 90% of graduates.^6-8^ Many factors may affect the decision to pursue subspecialization, including the opportunity to gain further experience leading to improved surgical competency. Thus, it is of great interest to characterize the potential association between orthopaedic surgeon subspecialty and outcomes for surgeries performed across subspecialties.^5,9-12^

A relatively recent study used the American Board of Orthopaedic Surgeons (ABOS) database to assess potential relationships between orthopaedic subspecialization and hip fracture outcomes.^13^ This study found no major differences in overall risk of surgical complications for hip fractures based on fellowship training. Limitations to the database used in this study was that only early-career surgeons were included and length of follow-up was limited and self-reported. In addition, the database lacks the ability to extract patient factors such as comorbidities, which are known to influence outcomes analyses.^14,15^

This study sought to leverage a large, national insurance claims database to assess the potential correlation of orthopaedic surgeon subspecialty with surgical outcomes following geriatric femoral neck fractures. Perioperative medical and five-year surgical outcome metrics were assessed.

## Methods

### Study Cohorts

This study used the 2016 to 2022 M161Ortho PearlDiver Mariner Patient Claims Database (PearlDiver Technologies, Colorado Springs, CO, USA).^16^ This is a national administrative data set that has been widely used for hip fracture research.^17–22^ As data output from this database are deidentified and presented only in aggregate form, our institutional review board determined studies using this database to be exempt from review.

Patients were included if they were aged 65 years or older and had a diagnosis of femoral neck fracture identified using ICD-10 codes. Surgical treatment was confirmed using Current Procedural Terminology (CPT) codes corresponding to hemiarthroplasty (CPT-27236 and CPT-27125), total hip arthroplasty (CPT-27130), or percutaneous pinning (CPT-27235). To ensure adequate follow-up for outcome assessment, patients were required to have at least 90 days of postoperative data available. Patients were excluded if they were younger than 65 years old, presented with concurrent neoplastic or infectious diagnoses at the time of fracture, or had less than 90 days of follow-up. This approach allowed us to focus on a geriatric population undergoing standard surgical interventions with sufficient follow-up to evaluate short- and long-term outcomes.

Patients were then divided into cohorts based on the subspecialty of the operating orthopaedic surgeon. This was defined based on the corresponding three-letter orthopaedic subspecialty code of the operating provider encoded in the Mariner161 database for each patient (OAR: arthroplasty, OTA: trauma, all other orthopaedic subspecialty codes: nonarthroplasty/nontrauma).

### Adverse Events

Ninety-day adverse events were identified using ICD-10 coding. Adverse events were tracked both independently and in aggregate groups.

Aggregated serious adverse events (SAE) were noted if there was the occurrence of one of the following: sepsis, cardiac events (myocardial infarction and cardiac arrest), pulmonary embolism (PE), deep vein thrombosis (DVT), or surgical site infection. Aggregated minor adverse events (MAE) were noted if there was the occurrence of one of the following: acute kidney injury (AKI), wound complications (wound dehiscence), hematoma, pneumonia, transfusion, or urinary tract infection (UTI). Aggregated any adverse events (AAE) were noted if there was the occurrence of a SAE or MAE. In addition, readmissions were tracked for 90 days postsurgery.

Five-year outcome metrics included five-year dislocation rates for hemiarthroplasty and total hip arthroplasty. Patients were tracked for time to first dislocation after the femoral neck fracture surgery using ICD codes for dislocation (ICD-10: S73.0X4X-S73.0X6X). Five-year implant survival rates were also calculated by tracking the patients across all three surgery types to first revision of the femoral neck fracture surgery, based on the appropriate CPT codes for revision (CPT-27134, CPT-27137, and CPT-27138).

### Statistical Analysis

The demographics of femoral neck surgeries, including age, sex, Elixhauser Comorbidity Index (ECI), and surgery type (hemiarthroplasty, total hip arthroplasty, pinning), were compared across the different orthopaedic subspecialties: nonarthroplasty/nontrauma, arthroplasty, and trauma. Chi-square tests were used for categorical variables (sex, age category, surgery type), and Student *t*-tests were applied for continuous variables (ECI).

Adverse events were compared between the surgical groups with univariable and multivariable analysis. Univariable analysis was performed using chi-square tests. Multivariable analysis was then done using multivariate logistic regression controlling for age, sex, and ECI.

Significance in univariable and multivariable analyses was adjusted for multiple comparisons using Bonferroni correction, with an adjusted *P* value of *P* < 0.007. For all other analyses, significance was defined as *P* < 0.05.

Time to dislocation up to 5 years was compared using Kaplan-Meier survival analysis and log-rank test for HA and THA. Implant survival until revision within five years postsurgery was also analyzed using Kaplan-Meier survival analysis and log-rank test across different orthopaedic subspecialties for each surgery type.

All statistical analyses were performed using Pearldiver's RSuite software (Pearldiver Technologies, Colorado Springs, CO, USA). Figures were created using GraphPad Prism 10 (GraphPad Software, San Diego, CA).

## Results

### Study Cohorts

A total of 150,728 surgeries were identified, which were formed by nontrauma/nonarthroplasty surgeons for 140,850, arthroplasty surgeons for 5,013, and trauma surgeons for 4,865. Characteristics of these patients are shown in Table 1.

**Table 1:** Demographics of Femoral Neck Fracture Surgery Patients Organized by Operating Surgeon Subspecialty

	Non-Arthroplasty, Non-Trauma	Arthroplasty	Trauma	*P* ^a^
Total (n)	140,850	5013	4865	
Age (mean ± SD), yr	75.39 ± 3.92	75.80 ± 4.23	76.24 ± 4.13	<0.001
65–69	10,291 (7.3%)	421 (8.4%)	342 (7.0%)	
70–74	45,568 (32.4%)	1217 (24.3%)	1071 (22.0%)	
75–79	53,342 (37.9%)	1843 (36.8%)	1885 (38.7%)	
>80	22,931 (16.3%)	992 (19.8%)	1088 (22.4%)	
Sex				<0.001
Female	93,979 (71.1%)	3127 (69.9%)	2989 (68.2%)	
Male	38,153 (28.9%)	1346 (30.1%)	1397 (31.8%)	
ECI (mean ± SD)	6.06 ± 3.95	6.50 ± 4.05	6.73 ± 4.12	<0.001
Surgery type				<0.001
Hemiarthroplasty	101,646 (71.3%)	3087 (60.5%)	3481 (70.9%)	
Total hip arthroplasty	18,225 (12.8%)	1435 (28.1%)	376 (7.7%)	
Pinning	22,649 (15.9%)	581 (11.4%)	1056 (21.5%)	

a Significance defined as *P* < 0.05.

Nonarthroplasty/nontrauma surgeons operated on slightly younger patients compared with arthroplasty surgeons and trauma surgeons (75.39 ± 3.92, 75.80 ± 4.23, and 76.24 ± 4.13 years old, respectively, *P* < 0.001, Table 1). Most patients for subspecialties were female (71.1%, 69.9%, and 68.2%, respectively, *P* < 0.001). ECI was lowest for the nontrauma/nonarthroplasty surgeons (6.06 ± 3.95, 6.50 ± 4.05, and 6.73 ± 4.12, respectively, *P* < 0.001).

Arthroplasty surgeons performed THA at a higher rate than trauma or nonarthroplasty/nontrauma surgeons (28.1% versus 7.7% and 12.8%, respectively, *P* < 0.001).

### Ninety-Day Outcomes

The univariate analyses for HA 90-day outcomes are shown in Table 2. This revealed statistically significant but clinically relatively similar rates of any adverse events, with nonarthroplasty/nontrauma surgeons demonstrating the lowest rate of any adverse events (39.6%) compared with arthroplasty (40.8%) and trauma surgeons (42.4%, *P* = 0.002). Statistically significant but again clinically relatively similar findings were noted for serious adverse events (nonarthroplasty/nontrauma: 12.6%, arthroplasty: 13.6%, trauma 16.2%, *P* < 0.001). In addition, minor differences were observed for sepsis, AKI, wound dehiscence, and readmissions (all within 4% of each other across the subspecialties.

**Table 2:** Univariate Comparison for Hemiarthroplasty Between Cohorts Organized by Operating Surgeon Subspecialty

	Nonarthroplasty, Nontrauma	Arthroplasty	Trauma	*P* ^a^
AAE	39,590 (39.6%)	1221 (40.8%)	1458 (42.4%)	0.002
SAE	12,637 (12.6%)	412 (13.6%)	558 (16.2%)	<0.001
Sepsis	3861 (3.9%)	146 (4.9%)	196 (5.7%)	<0.001
Cardiac	2736 (2.7%)	95 (3.2%)	122 (3.6%)	0.183
PE	1950 (1.9%)	68 (2.3%)	87 (2.5%)	0.027
DVT	4556 (4.6%)	140 (4.7%)	179 (5.2%)	0.188
Surgical site infections	1422 (1.4%)	31 (1.0%)	55 (1.6%)	0.120
MAE	34,852 (34.8%)	1080 (36.1%)	1263 (36.8%)	0.028
AKI	9436 (9.4%)	338 (11.3%)	413 (12.0%)	<0.001
Wound dehiscence	899 (0.9%)	35 (1.2%)	53 (1.5%)	<0.001
Hematoma	959 (1.0%)	26 (0.9%)	35 (1.0%)	0.8229
Pneumonia	8935 (8.9%)	246 (8.2%)	336 (9.8%)	0.085
Transfusion	6500 (6.5%)	200 (6.7%)	193 (5.6%)	0.108
UTI	20,219 (20.2%)	533 (17.8%)	707 (20.6%)	0.072
Readmission	15,394 (15.4%)	531 (17.7%)	621 (18.1%)	<0.001

AAE = any adverse event, SAE = serious adverse event, PE = pulmonary embolism, DVT = deep vein thrombosis, MAE = major adverse event, AKI = acute kidney injury, UTI = urinary tract infection

a Significance defined as *P* < 0.007 based on Bonferroni correction.

The univariate analysis for THA 90-day outcomes revealed no notable differences in any 90-day adverse events or readmissions among subspecialties (*P* > 0.007 for all, Table 3). The univariate analysis for percutaneous pinning outcomes also revealed no notable differences in 90-day adverse events or readmissions among subspecialties (*P* > 0.007 for all, Table 4).

**Table 3:** Univariate Comparison for Total Hip Arthroplasty Between Cohorts Organized by Operating Surgeon Subspecialty

	Nonarthroplasty, Nontrauma	Arthroplasty	Trauma	*P* ^a^
AAE	6064 (33.3%)	476 (33.1%)	138 (36.7%)	0.371
SAE	2148 (11.8%)	178 (12.4%)	58 (15.4%)	0.082
Sepsis	544 (3.0%)	40 (2.8%)	18 (4.8%)	0.113
Cardiac	367 (2.0%)	28 (2.0%)	13 (3.5%)	0.142
PE	362 (2.0%)	39 (2.7%)	12 (3.2%)	0.052
DVT	856 (4.7%)	71 (5.0%)	20 (5.3%)	0.790
Surgical site infections	294 (1.6%)	17 (1.2%)	5 (1.3%)	0.322
MAE	5142 (28.2%)	384 (26.7%)	114 (30.3%)	0.301
AKI	1337 (7.3%)	113 (7.9%)	34 (9.0%)	0.363
Wound dehiscence	228 (1.3%)	20 (1.4%)	10 (2.7%)	0.053
Hematoma	233 (1.3%)	12 (0.83%)	3 (0.8%)	0.252
Pneumonia	1061 (5.8%)	80 (5.6%)	25 (6.7%)	0.725
Transfusion	1337 (7.3%)	100 (7.0%)	24 (6.4%)	0.686
UTI	2668 (14.6%)	193 (13.4%)	59 (15.7%)	0.373
Readmission	2909 (16.0%)	243 (16.9%)	78 (21.0%)	0.031

AAE = any adverse event, SAE = serious adverse event, PE = pulmonary embolism, DVT = deep vein thrombosis, MAE = major adverse event, AKI = acute kidney injury, UTI = urinary tract infection

a Significance defined as *P* < 0.007 based on Bonferroni correction.

**Table 4:** Univariate Comparison for Percutaneous Pinning Between Cohorts Organized by Operating Surgeon Subspecialty

	Nonarthroplasty, Nontrauma	Arthroplasty	Trauma	*P* ^a^
AAE	7119 (31.4%)	193 (33.2%)	332 (31.4%)	0.657
SAE	2142 (9.5%)	69 (11.9%)	123 (11.7%)	0.011
Sepsis	689 (3.0%)	18 (3.1%)	40 (3.8%)	0.390
Cardiac	488 (2.2%)	15 (2.6%)	34 (3.2%)	0.059
PE	358 (1.6%)	14 (2.4%)	17 (1.6%)	0.291
DVT	772 (3.4%)	22 (3.8%)	38 (3.6%)	0.842
Surgical site infections	145 (0.6%)	4 (0.7%)	14 (1.3%)	0.059
MAE	6239 (27.6%)	165 (28.4%)	279 (26.4%)	0.646
AKI	1699 (7.5%)	46 (7.9%)	92 (8.7%)	0.330
Wound dehiscence	79 (0.4%)	0 (0%)	6 (0.6%)	0.175
Hematoma	129 (0.6%)	5 (0.9%)	10 (1.0%	0.206
Pneumonia	1650 (7.3%)	49 (8.4%)	70 (6.6%)	0.405
Transfusion	700 (3.1%)	17 (2.9%)	25 (2.4%)	0.404
UTI	3850 (17.0%)	96 (14.8%)	169 (16.0%)	0.376
Readmission	2609 (11.5%)	78 (13.4%)	143 (13.5%)	0.054

AE = any adverse event, SAE = serious adverse event, PE = pulmonary embolism, DVT = deep vein thrombosis, MAE = major adverse event, AKI = acute kidney injury, UTI = urinary tract infection

a Significance defined as *P* < 0.007 based on Bonferroni correction.

The multivariate analysis for HA 90-day outcomes demonstrates nuanced differences among surgeon cohorts (Table 5). Trauma surgeons, in comparison to the reference group, exhibited an increased the risk of SAE (OR: 1.15 [1.06 to 1.26], *P* = 0.001) and a lower risk of transfusion (OR: 0.80 [0.69 to 0.92], *P* = 0.002). No other notable differences in adverse events were found between surgeon cohorts.

**Table 5:** Multivariate Comparison for Hemiarthroplasty Between Cohorts Organized by Operating Surgeon Subspecialty

	Nonarthroplasty, Nontrauma	Arthroplasty, OR (95% CI)	*P* ^a^	Trauma, OR (95% CI)	*P* ^a^
AAE	Ref	0.97 (0.90-1.05)	0.452	0.98 (0.91-1.04)	0.465
SAE	Ref	1.05 (0.95-1.16)	0.307	1.15 (1.06-1.26)	0.001
Sepsis	Ref	1.17 (0.96-1.42)	0.100	1.23 (1.04-1.45)	0.014
Cardiac	Ref	1.23 (0.89-1.71)	0.205	1.05 (0.78-1.44)	0.758
PE	Ref	1.10 (0.78-1.54)	0.600	1.28 (0.95-1.71)	0.103
DVT	Ref	1.14 (0.86-1.52)	0.349	1.31 (1.03-1.66)	0.025
Surgical site infections	Ref	0.53 (0.33-0.84)	0.008	1.00 (0.74-1.37)	0.972
MAE	Ref	0.96 (0.89-1.03)	0.280	0.96 (0.89-1.02)	0.207
AKI	Ref	1.06 (0.95-1.19)	0.266	1.05 (0.95-1.15)	0.372
Wound dehiscence	Ref	1.19 (0.86-1.64)	0.300	1.43 (1.09-1.87)	0.009
Hematoma	Ref	0.74 (0.47-1.16)	0.187	0.81 (0.55-1.21)	0.307
Pneumonia	Ref	0.89 (0.79-1.00)	0.053	0.96 (0.86-1.06)	0.404
Transfusion	Ref	0.84 (0.72-0.98)	0.028	0.80 (0.69-0.92)	0.002
UTI	Ref	0.33 (0.22-0.51)	0.031	0.91 (0.84-0.99)	0.030
Readmission	Ref	1.07 (0.97-1.18)	0.155	1.06 (0.97-1.15)	0.201

ref = reference group, AAE = any adverse event, SAE = serious adverse event, PE = pulmonary embolism, DVT = deep vein thrombosis, MAE = major adverse event, AKI = acute kidney injury, UTI = urinary tract infection, OR = odds ratio, CI = confidence interval

a Significance defined as *P* < 0.007 based on Bonferroni correction.

The multivariate analysis for THA 90-day outcomes compared the three surgeon cohorts and revealed no notable differences were observed in 90-day adverse events/severe adverse events or readmission rates among subspecialty cohorts (*P* > 0.007, Table 6). The multivariate analysis for PP outcomes revealed arthroplasty surgeons had a lower risk of UTIs compared with the reference group (OR: 0.68 [0.51 to 0.89], *P* < 0.001). Trauma surgeons, compared with the reference group, demonstrated a reduced risk of MAE (OR: 0.79 [0.68 to 0.93], *P* = 0.004, Table 7). No other notable differences were noted between subspecialties in all other outcomes.

**Table 6:** Multivariate Comparison for Total Hip Arthroplasty Between Cohorts Organized by Operating Surgeon Subspecialty

	Nonarthroplasty, non-trauma	Arthroplasty, OR (95% CI)	*P* ^a^	Trauma, OR (95% CI)	*P* ^a^
AAE	Ref	0.87 (0.76-1.00)	0.040	0.85 (0.65-1.11)	0.245
SAE	Ref	0.95 (0.78-1.15)	0.589	1.15 (0.78-1.63)	0.463
Sepsis	Ref	0.84 (0.56-1.21)	0.377	1.31 (0.64-2.36)	0.421
Cardiac	Ref	0.93 (0.57-1.41)	0.735	1.18 (0.46-2.45)	0.693
PE	Ref	1.15 (0.75-1.70)	0.498	1.60 (0.71-3.04)	0.203
DVT	Ref	1.06 (0.79-1.40)	0.681	0.78 (0.39-1.39)	0.442
Surgical site infections	Ref	0.61 (0.31-1.06)	0.107	0.83 (0.26-1.97)	0.717
MAE	Ref	0.82 (0.71-0.95)	0.008	0.86 (0.65-1.14)	0.318
AKI	Ref	0.97 (0.76-1.23)	0.819	1.07 (0.65-1.67)	0.780
Wound dehiscence	Ref	0.97 (0.52-1.64)	0.910	2.16 (1.01-4.66)	0.048
Hematoma	Ref	0.64 (0.31-0.95)	0.196	0.60 (0.16-1.88)	0.473
Pneumonia	Ref	0.78 (0.58-1.04)	0.010	0.92 (0.51-1.52)	0.754
Transfusion	Ref	0.89 (0.69-1.14)	0.377	0.60 (0.31-1.03)	0.084
UTI	Ref	0.81 (0.67-0.97)	0.024	0.90 (0.61-1.27)	0.566
Readmission	Ref	1.09 (0.92-1.28)	0.317	1.03 (0.72-1.42)	0.880

ref = reference group, AAE = any adverse event, SAE = serious adverse event, PE = pulmonary embolism, DVT = deep vein thrombosis, MAE = major adverse event, AKI = acute kidney injury, UTI = urinary tract infection, OR = odds ratio, CI = confidence interval

a Significance defined as *P* < 0.007 based on Bonferroni correction.

**Table 7:** Multivariate Comparison for Percutaneous Pinning Between Cohorts Organized by Operating Surgeon Subspecialty

	Nonarthroplasty, nontrauma	Arthroplasty, OR (95% CI)	*P* ^a^	Trauma, OR (95% CI)	*P* ^a^
AAE	Ref	0.82 (0.67-1.01)	0.069	0.85 (0.73-0.98)	0.030
SAE	Ref	0.96 (0.69-1.31)	0.808	1.07 (0.86-1.32)	0.531
Sepsis	Ref	1.00 (0.57-1.63)	0.997	0.78 (0.50-1.16)	0.252
Cardiac	Ref	0.93 (0.46-1.67)	0.836	1.43 (0.96-2.05)	0.063
PE	Ref	1.20 (0.56-2.20)	0.604	0.78 (0.41-1.37)	0.410
DVT	Ref	0.74 (0.40-1.25)	0.300	0.99 (0.67-1.40)	0.940
Surgical site infections	Ref	0.63 (0.10-2.00)	0.522	1.82 (0.93-3.22)	0.057
MAE	Ref	0.82 (0.66-1.02)	0.080	0.79 (0.68-0.93)	0.004
AKI	Ref	0.86 (0.59-1.22)	0.416	1.00 (0.77-1.27)	0.998
Wound dehiscence	Ref	0.00 (0.00-33.00)	0.977	1.51 (0.53-3.37)	0.377
Hematoma	Ref	1.47 (0.45-3.52)	0.449	1.52 (0.68-2.92)	0.255
Pneumonia	Ref	1.22 (0.87-1.66)	0.241	0.75 (0.56-0.98)	0.042
Transfusion	Ref	0.80 (0.43-1.38)	0.464	0.53 (0.31-0.85)	0.014
UTI	Ref	0.68 (0.51-0.89)	<0.001	0.80 (0.66-0.96)	0.022
Readmission	Ref	1.01 (0.75-1.33)	0.964	0.93 (0.75-1.15)	0.512

ref = reference group, AAE = any adverse event, SAE = serious adverse event, PE = pulmonary embolism, DVT = deep vein thrombosis, MAE = major adverse event, AKI = acute kidney injury, UTI = urinary tract infection, OR = odds ratio, CI = confidence interval

a Significance defined as *P* < 0.007 based on Bonferroni correction.

### Five-Year Dislocations and Revisions

**Figure 1:**
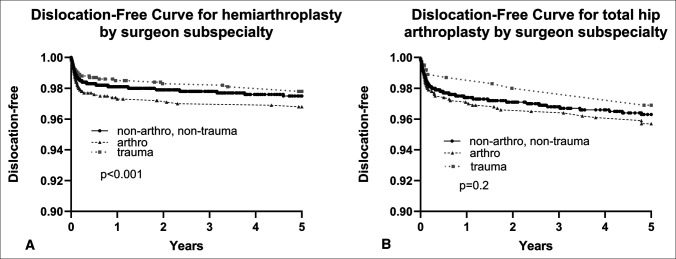
Five-year Kaplan-Meier curve for time to dislocation for geriatric femoral neck fracture patients who received hemiarthroplasty (**A**) or total hip arthroplasty (**B**), compared by subspecialty of operating orthopaedic surgeon.

Dislocation rate was compared by subspecialty. Arthroplasty surgeons demonstrated a higher risk of dislocation for HA implants (*P* < 0.001, Figure 1). However, the difference was clinically similar, as the absolute dislocation-free rates between cohorts remained within 1% of each other (nonarthroplasty/nontrauma: 97.5%, arthroplasty 96.8%, trauma: 97.8%). No notable differences were observed between subspecialties for dislocation rates of THA implants (*P* = 0.2).

**Figure 2:**
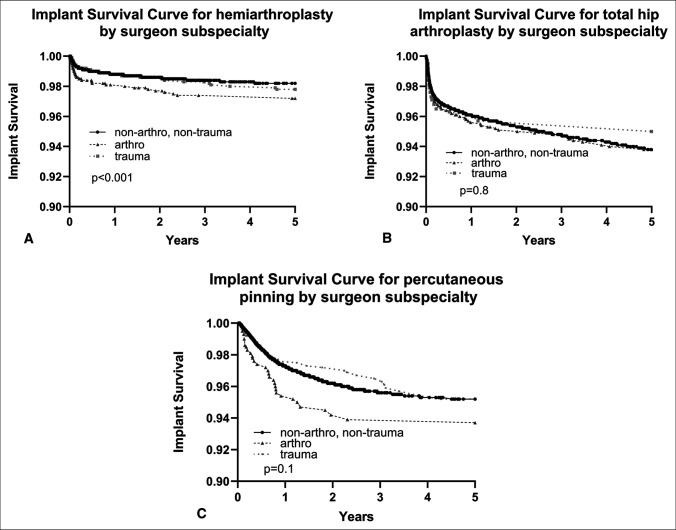
Five-year Kaplan-Meier curve of implant survival until revision for geriatric femoral neck fracture patients who received hemiarthroplasty (**A**), total hip arthroplasty (**B**), or percutaneous pinning (**C**), compared by subspecialty of operating orthopaedic surgeon.

Five-year implant survival until revision was compared by subspecialty. Although THA and PP showed no notable differences in survival (*P* > 0.05, Figure 2), arthroplasty surgeons exhibited slightly lower survival for HA implants (*P* < 0.001, Figure 2). However, the difference was again clinically similar, as the absolute survival rates between the cohorts remained within 1% of each other (nonarthroplasty/nontrauma: 98.2%, arthroplasty 97.2%, trauma: 97.8%).

## Discussion

Femoral neck fractures are common and may be managed by orthopaedic surgeons of different subspecialties. This study assessed the practice patterns and outcomes of nonarthroplasty/nontrauma, arthroplasty, and trauma orthopaedic surgeons in the management of these fractures.

The notable majority of these fractures were managed by nonarthroplasty/nontrauma orthopaedic surgeons. Arthroplasty surgeons performed THA at a higher rate than trauma and nonarthroplasty/nontrauma orthopaedic surgeons, consistent with the prior literature.^20^ This also suggests that arthroplasty surgeons are more likely to recommend THA than HA for hip fractures for older patients in general.

In terms of 90-day postoperative medical outcomes, the overarching finding of this study was similarity in outcomes for the surgeons of different subspecialties. For HA, this is in concordance with the current literature in which Schumaier et al^23^ concluded the same in 292 patients undergoing HA for displaced femoral neck fractures.

For THA, prior studies had reported conflicting findings. Ryan et al showed that among 291 hip fracture patients, 90-day adverse events were not different between arthroplasty versus other subspecialty orthopaedic surgeons.^24^ However, Thomas et al found that in 149 patients with femoral neck fractures undergoing THA, there were reduced major postoperative complications if the surgery was performed by arthroplasty surgeons compared with trauma surgeons.^25^ Another study by Mahure et al analyzed 16,882 total joint arthroplasties and found that fellowship-trained surgeons performed THAs with shorter surgical times, shorter hospital stays, higher rates of discharge to home, faster mobilization, and lower opioid requirements compared with nonfellowship-trained surgeons.^26^ With large statistical power (150,728 patients), this study provides an important contribution to the literature.

For PP, this study found no difference in 90-day outcomes following hip fracture managed by PP between surgeons of different subspecialties. However, to our knowledge, this study is the first to analyze such data.

In terms of 5-year rates of dislocation for those undergoing HA and THA, this study found all surgical subspecialty groups were within approximately 1.2% of each other. This study contributes to the conflicting literature on this topic as the first study to compare dislocation rates after five years of follow-up. Findings of this study are congruent with Thomas et al, which found no notable difference in dislocation rates between subspecialty groups within one year of surgery. However, Otteson et al reported that trauma surgeons had the lowest dislocation rate within the ABOS database.

In terms of 5-year rates of revision for those undergoing HA, THA, and PP, this study found that all surgical specialty groups were within approximately 1.2% of each other. Although Thomas et al found improved 1-year implant survival rate after THA for arthroplasty surgeons compared with others, there are no studies to our knowledge with five-year or longer revision rates comparing outcomes of these procedures by subspecialty training. However, DeAngelis et al recently reported that the 2-year risk of revision surgery after fixation of low-energy femoral neck fractures did not differ between fellowship-trained and non–fellowship-trained surgeons, based on an analysis of the FAITH trial database.^27^ This finding further supports the notion that surgeon subspecialty may not markedly affect revision surgery rates following hip surgeries in this patient population.

In general, surgeons of different subspecialities likely self-select for whom takes care of femoral neck fracture patients. The current large-cohort study found the 90-day and five-year results to be very similar across the different subspecialty groups studied. The similar outcomes between subspecialties for femoral neck fracture surgery also matches the aforementioned ABOS study, which found overall no major difference between subspecialties for hip fracture surgery.^13^

There are limitations to this study. First, as with all administrative database studies, the accuracy of the data used is dependent on coding available. Second, specifics of fracture morphology and clinical outcomes were not able to be assessed. Finally, we could not assess the specific surgical approaches, bearing diameters, or implant constructs used in the surgeries studied.

## Conclusion

Although surgeons of different subspecialties may perform hemiarthroplasty, total arthroplasty, or percutaneous pinning for femoral neck fractures, this study found overall similar results between the subspecialties in terms of 90-day adverse outcomes and five-year rates of revision/dislocation. These results are encouraging in that those who select to care for such fractures have overall comparable outcomes by the metrics assessed. Therefore, surgeons across various subspecialties can be reassured that their care for geriatric femoral neck fractures yields comparable outcomes, supporting flexible and multidisciplinary approaches to surgical management.

## References

[1] Cooper C: The crippling consequences of fractures and their impact on quality of life. Am J Med 1997;103:S12-S19. 10.1016/s0002-9343(97)90022-x 9302893

[2] Veronese N Maggi S: Epidemiology and social costs of hip fracture. Injury 2018;49:1458-1460. 29699731 10.1016/j.injury.2018.04.015

[3] Wilson JM Jones CA Holmes JS : Fixation vs arthroplasty for femoral neck fracture in patients aged 40-59 years: A propensity-score-matched analysis. Arthroplasty Today 2022;14:175-182. 35342781 10.1016/j.artd.2021.10.019 PMC8943217

[4] Florschutz AV Langford JR Haidukewych GJ Koval KJ: Femoral neck fractures: Current management. J Orthop Trauma 2015;29:121-129. 25635363 10.1097/BOT.0000000000000291

[5] Mead M Atkinson T Srivastava A Walter N: The return on investment of orthopaedic fellowship training: A ten-year update. J Am Acad Orthop Surg 2020;28:e524-e531. 31688369 10.5435/JAAOS-D-19-00276

[6] Horst PK Choo K Bharucha N Vail TP: Graduates of orthopaedic residency training are increasingly subspecialized: A review of the American Board of Orthopaedic Surgery part II database. J Bone Joint Surg Am 2015;97:869-875. 25995502 10.2106/JBJS.N.00995

[7] Daniels AH DiGiovanni CW: Is subspecialty fellowship training emerging as a necessary component of contemporary orthopaedic surgery education? J Grad Med Educ 2014;6:218-221. 24949124 10.4300/JGME-D-14-00120.1 PMC4054719

[8] American Board of Orthopaedic Surgery. Rules and Procedures for Residency Education, Part I, and Part II Examinations. Chapel Hill, NC, American Board of Orthopaedic Surgery, 2023. Accessed September 17, 2023.

[9] Almansoori KA Clark M: Increasing trends in orthopedic fellowships are not due to inadequate residency training. Educ Res Int 2015;2015:191470-191479.

[10] Silvestre J Wu HH Thompson TL Kang JD: Utility of spine surgery fellowship training for orthopaedic surgeons in the United States. J Am Acad Orthop Surg 2023;31:335-340. 36729747 10.5435/JAAOS-D-22-00788

[11] Yin B Gandhi J Limpisvasti O Mohr K ElAttrache NS: Impact of fellowship training on clinical practice of orthopaedic sports medicine. J Bone Joint Surg Am 2015;97:e27. 25740036 10.2106/JBJS.N.00164

[12] Matson AP Kavolus JJ Byrd WA Leversedge FJ Brigman BE: Influence of trainee experience on choice of orthopaedic subspecialty fellowship. J Am Acad Orthop Surg 2018;26:e62-e67. 29283897 10.5435/JAAOS-D-16-00701

[13] Ottesen TD Mercier MR Brand J Amick M Grauer JN Rubin LE: The case for decreased surgeon-reported complications due to surgical volume and fellowship status in the treatment of geriatric hip fracture: An analysis of the ABOS database. PLoS One 2022;17:e0263475. 35213546 10.1371/journal.pone.0263475 PMC8880652

[14] Feinstein AR: The pre-therapeutic classification of co-morbidity in chronic disease. J Chronic Dis 1970;23:455-468. 26309916 10.1016/0021-9681(70)90054-8

[15] Perka C Arnold U Buttgereit F: Influencing factors on perioperative morbidity in knee arthroplasty. Clin Orthop Relat Res 2000;378:183-191. 10.1097/00003086-200009000-00028 10986993

[16] PearlDiver Technologies: PearlDiver User Manual Version 2.7. Wellington, NZ, PearlDiver, 2021.

[17] Erickson BJ Nwachukwu BU Kiriakopoulos E : In-hospital mortality risk for femoral neck fractures among patients receiving medicare. Orthopedics 2015;38:e593-e596. 26186321 10.3928/01477447-20150701-57

[18] Kahlenberg CA Richardson SS Schairer WW Cross MB: Rates and risk factors of conversion hip arthroplasty after closed reduction percutaneous hip pinning for femoral neck Fractures-A population analysis. J Arthroplasty 2018;33:771-776. 29089225 10.1016/j.arth.2017.10.004

[19] Zhao AY Agarwal AR Harris AB Cohen JS Golladay GJ Thakkar SC: The association of prior fragility fractures on 8-year periprosthetic fracture risk following total hip arthroplasty. J Arthroplasty 2023;38:S265-S269.e5. 10.1016/j.arth.2023.02.043 36828052

[20] Gupta P Golub IJ Lam AA Diamond KB Vakharia RM Kang KK: Causes, risk factors, and costs associated with ninety-day readmissions following primary total hip arthroplasty for femoral neck fractures. J Clin Orthop Trauma 2021;21:101565. 34476176 10.1016/j.jcot.2021.101565 PMC8387745

[21] Gouzoulis MJ Joo PY Caruana DL Kammien AJ Rubio DR Grauer JN: Incidental durotomy after posterior lumbar decompression surgery associated with increased risk for venous thromboembolism. J Am Acad Orthop Surg 2023;31:e445-e450. 36727948 10.5435/JAAOS-D-22-00917

[22] Wu VJ Ross BJ Sanchez FL Billings CR Sherman WF: Complications following total hip arthroplasty: A nationwide database study comparing elective vs hip fracture cases. J Arthroplasty 2020;35:2144-2148.e3. 32229152 10.1016/j.arth.2020.03.006

[23] Schumaier AP Andrews EG Yue RA : Hemiarthroplasty for femoral neck fractures: Does surgeon subspecialty affect perioperative outcomes? J Orthop Trauma 2020;34:589-593. 33065659 10.1097/BOT.0000000000001839

[24] Ryan SP Padilla JA Schwarzkopf R Gage MJ Bolognesi MP Seyler TM: Arthroplasty surgeons do not improve acute outcomes for patients with hip fracture relative to other subspecialists. Orthopedics 2020;43:e442-e446. 32602917 10.3928/01477447-20200619-11

[25] Thomas JC Haidukewych GJ: Total hip arthroplasty for acute femoral neck fractures: Who should perform the operation-adult reconstructive or trauma surgeons? J Orthop Trauma 2021;35:606-611. 34050073 10.1097/BOT.0000000000002091

[26] Mahure SA Feng JE Schwarzkopf RM Long WJ: The impact of arthroplasty fellowship training on total joint arthroplasty: Comparison of peri-operative metrics between fellowship-trained surgeons and non-fellowship-trained surgeons. J Arthroplasty 2020;35:2820-2824. 32540307 10.1016/j.arth.2020.05.027

[27] DeAngelis RD Stein MK Minutillo GT ; FAITH Investigators: Subspecialty fellowship training is not associated with better outcomes in fixation of low-energy femoral neck fractures-an analysis of the fixation using alternative implants for the treatment of hip fractures database. J Orthop Trauma 2022;36:208-212. 34483325 10.1097/BOT.0000000000002264 PMC8882705

